# From Traditional Knowledge to SARS-CoV-2 Entry Inhibitor Metabolites: Ethnopharmacological Investigation of *Uncaria tomentosa* (Willd. ex Schult.) DC

**DOI:** 10.3390/plants15131998

**Published:** 2026-06-27

**Authors:** Beatriz Ribeiro Ferreira, Mariana Freire Campos, Sarah Beatriz F. Rodrigues, Ana Beatriz Lima, Simony Carvalho Mendonça, Crisálida M. Vilanova, Denise F. Coutinho, Diego Allonso, Flavia Maria M. Amaral, Suzana Guimarães Leitão

**Affiliations:** 1 Programa de Pós-Graduação em Biotecnologia Vegetal e Bioprocessos, Universidade Federal do Rio de Janeiro, Rio de Janeiro 21941-902, RJ, Brazil; 2 Faculdade de Farmácia, Universidade Federal do Rio de Janeiro, Rio de Janeiro 21941-902, RJ, Brazil; camposmariana@biof.ufrj.br (M.F.C.); abeatrizlima@ufrj.br (A.B.L.); simonycarvalho@farmacia.ufrj.br (S.C.M.); diegoars@biof.ufrj.br (D.A.); 3 Departamento de Farmácia, Universidade Federal do Maranhão, Campus Bacanga, São Luís 65080-805, MA, Brazil; sarah.bfr@discente.ufma.br (S.B.F.R.); crisalida.vilanova@ufma.br (C.M.V.); denise.coutinho@ufma.br (D.F.C.); flavia.amaral@ufma.br (F.M.M.A.)

**Keywords:** ethnopharmacology, medicinal plants, *Uncaria tomentosa*, COVID-19, SARS-CoV-2, LC-MS/MS, molecular networking, antiviral activity

## Abstract

The COVID-19 pandemic stimulated the widespread use of traditional medicinal plants in Brazil, particularly in regions with limited access to healthcare and approved therapies. In this context, medicinal plants became accessible alternatives for symptom management and disease prevention, highlighting the value of traditional health systems as sources of biologically relevant species for further investigation. This study documented medicinal plants used to prevent and treat COVID-19 in São Luís, Maranhão, Brazil, and evaluated the inhibitory activity of *Uncaria tomentosa* (Willd. Ex Schult)extracts, the most frequently cited species, against the SARS-CoV-2 RBD:ACE2 interaction. An ethnopharmacological survey was initially conducted with 400 participants between November 2022 and March 2023 where a total of 38 medicinal ethnospecies were reported, with an overall prevalence of medicinal plant use of 22.75%. Considering that aqueous preparations were the predominant form of use reported by participants, both aqueous and ethanolic extracts were prepared from the stem bark and leaves of *U. tomentosa* and evaluated in an in vitro Spike (RBD) inhibition assay. The highest inhibitory activity was observed for stem bark extracts of *U. tomentosa*, which achieved 98.09% inhibition for the ethanolic extract and 73.40% for the aqueous extract. Preparations obtained from the leaves showed lower activity, with inhibition values of 41.12% and 19.74%, respectively. Chemical profiling was performed by LC-MS/MS combined with molecular networking. Chemical analysis enabled the annotation of oxindole alkaloids and flavonoids previously reported in the literature as exhibiting relevant biological activities in models of viral infections. These findings highlight the ethnopharmacological relevance of *U. tomentosa*, support its potential as a source of bioactive metabolites, and reinforce the value of ethnopharmacological approaches in identifying promising species for further biological investigation.

## 1. Introduction

The COVID-19 pandemic, caused by Severe Acute Respiratory Syndrome Coronavirus 2 (SARS-CoV-2), was one of the greatest health crises in recent history. Initially, the rapid spread of the virus was compounded by the complete lack of specific pharmacological therapies, prompting the scientific community to focus its immediate efforts on drug repurposing and the urgent search for effective antiviral agents [1]. In parallel with this clinical movement, the overburdening of healthcare systems led the population to seek complementary strategies for the prevention and relief of primary symptoms. In this context of uncertainty, the use of medicinal plants and home remedies emerged as an accessible resource for basic care, particularly in regions with a long history of integrating traditional knowledge of local flora into daily health practices [2].

In Brazil, this practice is supported by a rich historical and cultural context and is institutionalized through public policies such as the National Policy on Integrative and Complementary Practices in the Brazilian unified health system (PNPIC-SUS) [3]. During the health crisis, national ethnopharmacological surveys recorded a significant increase in searches for plant species to manage symptoms associated with respiratory infection. However, in scientific literature, a necessary conceptual distinction is established between popular emergency use and formally validated therapeutic evidence. Citation data regarding the use of a plant species do not, by themselves, constitute proof of efficacy or antiviral potential; however, such records act as fundamental ethnopharmacological indicators for guiding and selecting targets in subsequent laboratory screenings [2,4].

In this context, the municipality of São Luís, the capital of Maranhão State in Brazil, constitutes a strategic field of study. As an urban center of intense circulation of people and knowledge and situated within an ecological transition region between the Amazon, Cerrado, and Caatinga biomes, the city hosts a rich plurality of therapeutic practices [4]. The investigation of the São Luís scenario fills an important geographical and documentary gap regarding how urban populations in the northeast mobilized local biodiversity to respond to the pandemic.

Among the species cited in this context, *Uncaria tomentosa* (Willd. ex Schult.) DC., commonly known as cat’s claw, stands out for its long history of use in managing inflammatory and infectious conditions. Scientific interest focuses on its profile of bioactive metabolites, with emphasis on pentacyclic and tetracyclic oxindole alkaloids, as well as polyphenols and proanthocyanidins [5]. However, laboratory investigation of the species requires rigor in the choice of raw material, since there is marked chemical variability among plant organs; the leaves and stem bark present distinct alkaloid profiles, a factor that directly influences the reproducibility of biological effects [5].

Coupled with its phytochemical complexity, the herbal medicine market faces severe quality control challenges, including adulteration and intentional substitution involving *Uncaria guianensis*, a species that shares similar morphology but has a divergent chemical composition [6]. Given these variables, scientific evidence on the real antiviral potential of *U. tomentosa*, specifically against SARS-CoV-2, remains scarce, underscoring the need for targeted biochemical studies to elucidate its preliminary mechanisms of action [7].

Beyond botanical and market classification, the prospection of potential therapeutic inputs requires an understanding of the molecular mechanisms of infection. One of the most robust biological targets is the initial stage of viral anchoring, mediated by the interaction between the receptor-binding domain (RBD) of the viral *Spike* protein and the angiotensin-converting enzyme 2 (ACE2) of host cells [8]. Biochemical assays capable of evaluating the blockade of this binding operate as an initial screening [9]. It is imperative to highlight, however, that the inhibition of this interaction in vitro indicates only a preliminary biochemical mechanism of interference with viral entry, and does not constitute proof of replication inhibition within the cell or of clinical efficacy.

Based on this panorama, the present study relies on the hypothesis that the plant species most frequently cited by the population of São Luís for the management of COVID-19 contain metabolites capable of modulating the initial stages of viral infection. Thus, this study aimed to document the medicinal plants used by residents of São Luís during the pandemic and, integrating ethno-knowledge with laboratory investigation, to perform the chemical characterization and biological evaluation of the RBD:ACE2 interaction blockade by the most prominent species in the survey, *Uncaria tomentosa*.

## 2. Results

### 2.1. Prevalence and Sociodemographic Profile

Of the 400 participants interviewed in the city of São Luís, Brazil, the overall prevalence of medicinal plant use for the treatment and/or prevention of COVID-19 was 22.75%. Medicinal plant use was more prevalent among women (25.1%) compared to men (17.4%). Furthermore, the prevalence of use increased significantly with age, being more common among adults aged 30–59 years (30.7%) and reaching its highest rate among those aged ≥ 60 years (42.9%). The association between age and medicinal plant use was statistically significant (*p* < 0.001). Regarding the sociodemographic characteristics of the users, the majority reported having completed high school (41.5%) and earning a monthly income of 2–4 times the minimum wage (40.5%).

### 2.2. Health Conditions and Concurrent Use

When asked about prior SARS-CoV-2 infection, 221 participants (55.25%) reported a positive test result. An additional 110 individuals (27.5%) reported experiencing symptoms consistent with COVID-19 but were not tested. In contrast, 69 participants (17.25%) reported neither symptoms nor a positive test.

Significantly, among participants who reported comorbidities, 39.56% were concurrently using synthetic medications and medicinal plants. Despite this high frequency of combined use, only 31.68% of these individuals had informed their physicians about the simultaneous use of medicinal plants. These findings suggest that potential herb–drug interactions may occur without adequate clinical monitoring among individuals with pre-existing health conditions, who are more likely to rely on conventional pharmacological treatments. The low rate of communication with healthcare professionals reveals an important gap in patient care and reinforces the need for greater awareness regarding the safe use of medicinal plants. Furthermore, these results highlight the importance of strengthening pharmacovigilance strategies and improving communication between patients and healthcare professionals to minimize the risks associated with concomitant use of medicinal plants and conventional medicines [10,11].

### 2.3. Sources of Information and Methods of Acquisition

Family members and friends were the primary sources of information regarding medicinal plant use (63%), followed by media (30%) and healthcare professionals (7%). Most of the plants were obtained from home gardens and backyards (55%) or local markets (33%), reflecting the centrality of traditional knowledge in transmitting this information [12,13,14].

### 2.4. Cited Ethnospecies and Preparation Methods

A total of 38 medicinal ethnospecies were mentioned (Table 1). The most cited was unha-de-gato/cat’s claw (*n* = 39), followed by boldo (*n* = 38), limão/lemon (*n* = 23), gengibre/ginger (*n* = 22), and alho/garlic (*n* = 16). Decoction was the most commonly used method of preparation (71.4%), particularly as teas.

### 2.5. Ethnobotanical Analysis and Highlighted Ethnospecies

**Figure 1:**
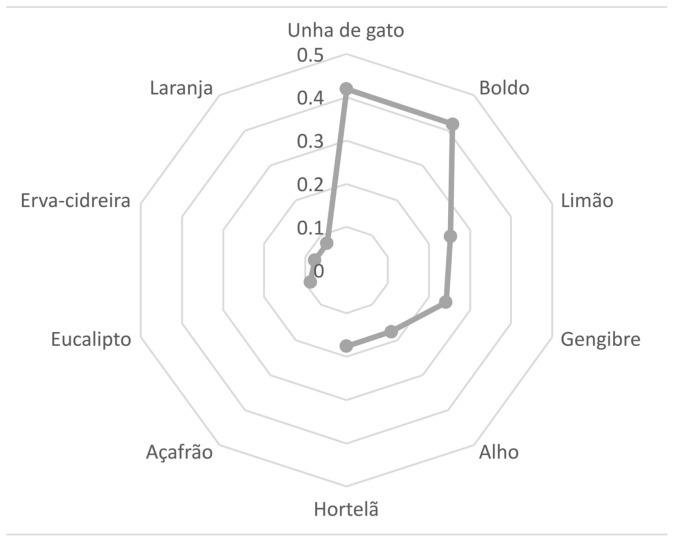
Relative frequency of citation (RFC) of the ten most frequently reported medicinal ethnospecies.

The six most cited ethnospecies also showed the highest values of importance (IVs) and consensual use (UCs), with Unha de gato (IVs = 0.2387; UCs = 0.8787) and Boldo (IVs = 0.2307; UCs = 0.8444) standing out (Figure 1). However, the frequency of popular use does not guarantee proven pharmacological efficacy. Clinical studies remain scarce, necessitating rigorous scientific evidence [13].

### 2.6. Inhibition of Spike(RBD):ACE2 Interaction by Uncaria tomentosa Extracts

*Uncaria tomentosa* was selected for further investigation due to its extensive history of biological activities, particularly its immunomodulatory and antiviral-related properties, as well as its status as the most frequently cited species in the ethnopharmacological survey. Given that the community predominantly reported using aqueous preparations, especially decoctions, both aqueous and ethanolic extracts from leaves and stem bark were evaluated. The inclusion of ethanolic extracts was justified by their widespread use in phytochemical investigations to optimize the extraction of bioactive secondary metabolites, thereby enabling comparisons with preparations more closely associated with traditional use. As part of an initial screening strategy, *U. tomentosa* extracts were evaluated at a single concentration (250 µg/mL) to identify samples with potential activity against the SARS-CoV-2 RBD:ACE2 interaction.

In vitro assays revealed that at 250 μg/mL, the stem bark extracts exhibited a greater capacity to inhibit RBD:ACE2 interaction, achieving inhibition rates of 98.09% (±0.39) and 73.40% (±0.31) for the ethanolic and aqueous extracts, respectively. In contrast, leaf extracts showed lower activity, with inhibition values of 41.12% (±0.12) for the ethanolic extract and 19.74% (±0.26) for the aqueous extract. Although the observed activity cannot be directly attributed to specific metabolites, the higher inhibitory potential of the stem bark extracts is consistent with previous reports describing this plant organ as a rich source of oxindole alkaloids, including mitraphylline and pteropodine, compounds frequently associated with the biological activities reported for the species [14].

### 2.7. Fingerprints by LC-MS/MS and Dereplication Using Molecular Networking

**Figure 2:**
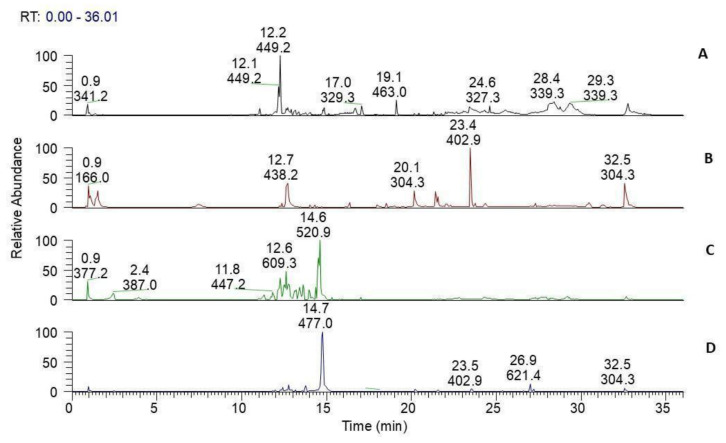
Aligned chromatograms of *U. tomentosa* ethanolic extracts by LC-ESI (+/−)-MS/MS ((**A**) Leaf extract in positive mode, (**B**) Leaf extract in negative mode, (**C**) Stem bark extract in positive mode, (**D**) Stem bark extract in negative mode).

High-Performance Liquid Chromatography coupled with tandem Mass Spectrometry (LC-MS/MS) of both leaf and stem bark ethanolic extracts of *Uncaria tomentosa* revealed distinct chemical profiles (Figure 2). Among the characterized compounds, indole alkaloids were annotated, including oxindole alkaloids (tetracyclic and pentacyclic indoloquinolizidine types). Oxindole alkaloids are considered important chemical markers of *Uncaria tomentosa* and *Uncaria guianensis*, being widely reported as characteristic constituents of these species.

**Figure 3:**
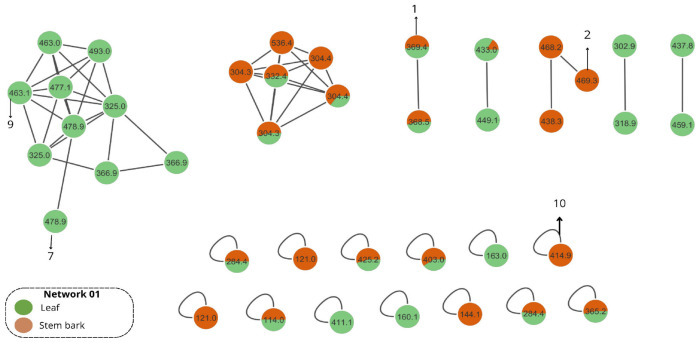
Feature-Based Molecular Network (FBMN) of the ethanolic extract from the stem bark and leaves of *Uncaria tomentosa*, in positive ion mode (Network 1). Green nodes represent ions detected in leaf extracts, whereas red nodes represent ions detected in stem bark extracts. The numbers correspond to the compounds listed in Table 2.

**Figure 4:**
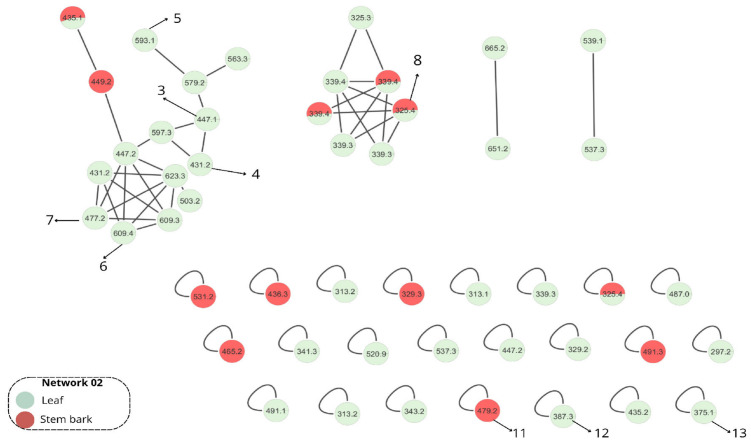
Feature-Based Molecular Network (FBMN) of the ethanolic extract from the stem bark and leaves of *Uncaria tomentosa*, in negative ion mode (Network 2). Green nodes represent ions detected in leaf extracts, whereas red nodes represent ions detected in stem bark extracts. The numbers correspond to the compounds listed in Table 2.

Molecular networks were constructed using the Feature-Based Molecular Network (FBMN) workflow. In the visual representation, the ions found exclusively in the stem bark are highlighted in brown (positive ion mode) and red (negative ion mode). These colors correspond to ions found specifically in the stem bark (Figure 3 and Figure 4).

The annotated compounds are indicated by their respective numbers in Table 2. Since the automated GNPS library search yielded no hits, structural annotations were performed manually. These proposals were based on a customized database compiled from an extensive literature review of the target species, coupled with a comprehensive analysis of MS/MS fragmentation profiles. Furthermore, the molecular networking approach facilitated chemical class-level annotations. Because structurally related compounds co-cluster due to similar fragmentation patterns, this strategy enabled the detection of other congeners within the same chemical class, which may further contribute to the observed biological activity.

Analysis of ions present in leaf and stem barkethanolic extracts revealed significant compositional differences, with well-defined clusters indicating distinct chemical profiles [15]. Table 2 summarizes the LC-MS/MS data of annotated compounds in the molecular network acquired in positive and negative ion modes.

**Figure 5:**
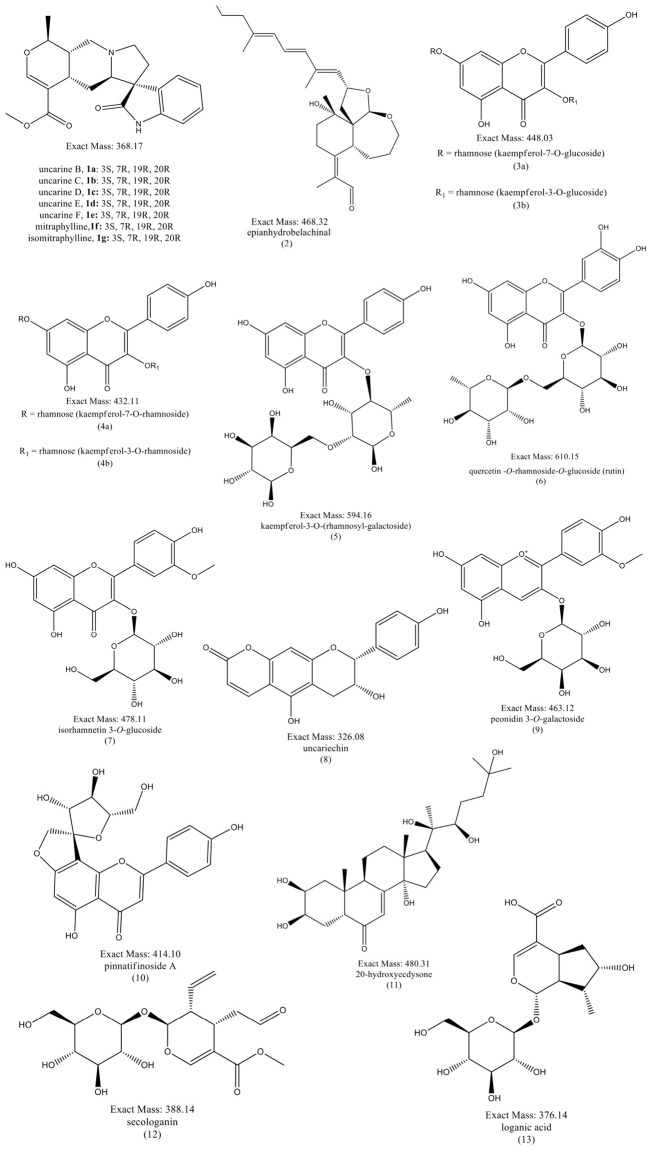
Chemical structures of the compounds annotated by LC-MS/MS in the ethanolic extracts of the leaves and stem bark of *U. tomentosa*.

The parent ion with *m*/*z* 369.4 [M + H]^+^ and its MS^2^ fragments may correspond to oxindole alkaloids that are structural isomers, such as uncarine B/C/D/E/F, mitraphylline, and isomitraphylline (**1a**–**g**) (Figure 5). Oxindole alkaloids are widely recognized as characteristic constituents and important chemical markers of *Uncaria tomentosa* and related species of the genus *Uncaria*.

In addition to alkaloids, several flavonoids were putatively annotated. These were assigned as flavonol derivatives with MS/MS profiles consistent with kaempferol-*O*-glucoside, kaempferol-*O*-rhamnoside, kaempferol-3-*O*-rhamnosyl-galactoside, quercetin-*O*-rhamnoside-*O*-glucoside (rutin), and isorhamnetin-*O*-glucoside. Other phenolic compounds were tentatively identified as the flavan-3-ol uncariechin and the anthocyanidin peonidin 3-*O*-galactoside. While MS/MS fragmentation patterns accurately identify neutral losses of hexose or deoxyhexose residues, they cannot distinguish specific sugar isomers. Therefore, precise carbohydrate identities (e.g., glucoside, rhamnoside) were proposed based on previous phytochemical reports for this species and the annotated records in the custom database. Monoterpene iridoids such as pinnatifinoside A, secologanin, and loganic acid were also detected, alongside the ecdysteroid 20-hydroxyecdysone. The possible presence of these metabolite classes highlights the chemical diversity of *U. tomentosa* extracts and is consistent with previous phytochemical studies that describe alkaloids, flavonoids, and iridoids as major constituents of this species (Figures S1–S13).

## 3. Discussion

During the COVID-19 pandemic, demand for alternative therapies increased across populations, particularly in contexts where conventional therapeutic options were limited or uncertain. In this scenario, the use of medicinal plants gained greater visibility and was frequently incorporated into healthcare strategies adopted by the population. Among these species, *Uncaria tomentosa* (cat’s claw) stands out, as it has been widely described in the literature for its therapeutic applications in several health conditions [15]. In this context, ethnopharmacological studies play a crucial role in bridging traditional knowledge and scientific investigation, enabling not only the identification of species with therapeutic potential but also the evaluation of risks associated with their use and the strengthening of pharmacovigilance strategies.

In the present study, *U. tomentosa* was the most frequently cited medicinal plant, being reported by 39 participants. This corresponded to 9.75% of the total sample and approximately 42.9% of medicinal plant users. This finding highlights the relevance of this species within the local therapeutic context. Most participants reported using it due to its accessibility and the long-standing family traditions associated with its use. Similar patterns have been reported in previous studies, which indicate that ethnopharmacological practices tend to intensify during public health emergencies, particularly when therapeutic uncertainty or limited access to conventional treatments is observed [24].

The inhibitory activity observed for *U. tomentosa* in the present study reinforces its ethnopharmacological relevance and demonstrates the capacity of its extracts to interfere with the interaction between the SARS-CoV-2 *Spike* protein and the ACE2 receptor. Since this interaction represents a major step in viral entry into host cells, its inhibition has been widely used as an initial screening tool to identify bioactive extracts and compounds for further investigation. In this context, the stem bark extracts exhibited the highest inhibition percentages, reaching 98.09% for the ethanolic extract and 73.40% for the aqueous extract. Conversely, the leaf extracts showed values of 41.12% and 19.74%, respectively. These results indicate marked differences in biological activity between the evaluated plant parts.

Although Lumit^®^ does not directly assess viral infection, it serves as a useful screening platform for rapidly evaluating large sample collections and selecting those capable of affecting the *Spike*:ACE2 binding process, thereby prioritizing candidates for subsequent evaluation in cell-based assays. Thus, the activity observed in the present study should be regarded as preliminary evidence supporting the potential of *U. tomentosa* for further investigation [25].

When these findings are interpreted in light of the ethnobotanical data obtained in this study, an asymmetry is observed between local usage patterns and the recommendations described in official compendia. While these sources frequently indicate the stem bark as the primary medicinal part of the species, the participants predominantly reported using the leaves in aqueous preparations. From a conservation and sustainable management perspective, this preference may be an advantage, given that continuous harvesting of the stem bark can cause trunk girdling and compromise the specimen’s survival. On the other hand, the results obtained indicate that the bark extracts exhibited a greater capacity to interfere with the *Spike*-ACE2 (RBD) interaction, highlighting the importance of understanding how different plant organs can influence the observed biological outcomes.

The expressive inhibitory activity demonstrated by the aqueous extract of the stem bark (73.40%) is of significant scientific relevance, as this preparation mimics the traditional forms of use identified in the ethnobotanical survey. Although the ethanolic extracts exhibited higher potency, likely due to the optimization of the solubility profile of nonpolar compounds, the data indicate that the water-soluble fractions also possess the capacity to interfere with the *Spike*-ACE2 interaction robustly. This finding bridges the gap between the experimental approach and the ethnopharmacological context documented in this study, reinforcing the importance of evaluating preparations that reflect the species’ traditional use.

The commercial availability of the plant material may partially explain the discrepancy between local usage patterns and phytotherapeutic recommendations. All participants reported purchasing the plant from the same establishment specializing in natural products, where only the leaves were available for commercialization. This observation suggests that consumption patterns are shaped not only by traditional knowledge but also by the market availability of the plant material, a factor that can influence how the population utilizes the species.

Phytochemical characterization of the extracts by LC-MS/MS revealed the presence of oxindole alkaloids and flavonoids, classes of metabolites previously associated with immunomodulatory, anti-inflammatory, and antiviral activities [26]. In addition, leaf and stem bark extracts exhibited distinct metabolic profiles, suggesting that the origin of the plant tissue may influence chemical composition and, consequently, biological activity [27]. Differences in the distribution of secondary metabolites among plant tissues are common and often associated with specific ecological and physiological roles [28].

Mass spectrometry-based molecular networking analysis (FBMN via GNPS) allowed the dereplication and annotation of compounds potentially associated with antiviral activity [29,30]. In this context, ions detected in the stem bark extract showed a stronger association with the inhibition of the RBD–ACE2 interaction. However, the annotation of oxindole alkaloids exclusively by mass spectrometry presents certain limitations, since structurally related compounds such as speciophylline, uncarine D, and mitraphylline may exhibit similar fragmentation patterns. Therefore, complementary techniques such as nuclear magnetic resonance (NMR) are often required for definitive structural confirmation [14,31]. The fragmentation patterns of these alkaloids typically involve cleavage of C–N and C–C bonds, particularly between the pyrrole and quinolizidine rings [26,32].

Several compounds previously reported in *Uncaria tomentosa* have been associated with antiviral activity. Among them, the oxindole alkaloid speciophylline has shown promising results in molecular docking studies against COVID-19 targets, including the SARS-CoV-2 main protease (*3CLpro*), an enzyme essential for viral replication [15]. Inhibition of this protease interferes with the processing of viral polyproteins, which is required for the formation of functional viral particles.

In addition to alkaloids, flavonoids such as luteolin and its derivatives have demonstrated anti-inflammatory and antiviral properties in different experimental models [33,34,35]. These metabolites may act on both viral targets and host cellular components, including the SARS-CoV-2 *3CLpro* protease and the ACE2 receptor, thereby interfering with key stages of the viral infection cycle [36,37]. Similarly, other flavonoids such as quercetin and isorhamnetin have been described as modulators of viral entry mechanisms. Experimental and computational studies suggest that these compounds may interact with the receptor-binding domain of the *Spike* protein, reducing its affinity for the ACE2 receptor and consequently limiting viral entry into host cells [38,39].

It is important to note that the biological activity of plant extracts often results from complex interactions among multiple metabolites. Minor constituents may contribute substantially to the observed effects, while more abundant metabolites may act synergistically or display limited activity depending on the molecular context [40]. In this regard, alkaloids, flavonoids, and terpenoids frequently exhibit pharmacological activity even at low concentrations. In contrast, highly abundant compounds such as tannins or sugars are not always directly responsible for the observed biological effects [41,42,43].

Beyond pharmacological aspects, the increasing commercial demand for *Uncaria tomentosa* in international markets has intensified harvesting pressure on the species’ natural populations [44,45]. The extraction of stem bark and roots for medicinal purposes may represent a destructive practice when conducted indiscriminately, raising concerns regarding species conservation.

In Brazil, *Uncaria tomentosa* was included in the Brazilian Pharmacopeia Herbal Medicines Formulary, 1st edition [46], which provides technical guidance on its therapeutic use, including the indication of stem bark as the primary medicinal part. However, the species was not maintained in the 2nd edition of the Brazilian Pharmacopeia Herbal Medicines Formulary [47]. This change reflects challenges related to the standardization of plant raw materials, quality control, and the availability of robust scientific evidence to support its continued inclusion in official regulatory documents.

Taken together, the findings of the present study highlight the importance of integrating ethnopharmacological knowledge with phytochemical and biological investigations. Such approaches contribute to a more comprehensive understanding of medicinal species and may support evidence-based regulatory decisions. Furthermore, strengthening pharmacovigilance strategies is essential to ensure the safe and rational use of medicinal plants, particularly during public health emergencies, when the search for alternative therapies tends to intensify.

## 4. Materials and Methods

### 4.1. Study Area

**Figure 6:**
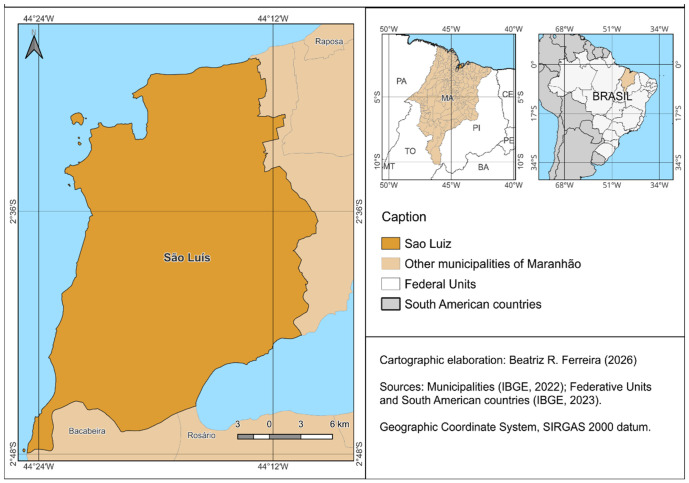
Geographic location of the municipality of São Luís, state of Maranhão, Brazil. Cartographic elaboration by the authors based on data from IBGE (2022) [49] and IBGE (2023) [50].

The study was conducted in São Luís, the capital of the State of Maranhão, located on Upaon-Açu Island, along the northeastern coast of Brazil (Figure 6). The city is part of the Brazilian Northeast region and is included within the Legal Amazon, constituting an ecological transition zone between the Amazon biome and the Cocais Forest, characterized by a humid tropical climate and high annual rainfall, conditions that favor high plant diversity [48]. According to the 2022 Demographic Census, São Luís is the most populous municipality in the state, with 1,037,775 inhabitants distributed over 583.063 km^2^, resulting in a population density of 1779.87 inhabitants/km^2^, and is consolidated as the main urban, political, and healthcare center of the region [48].

From a physical-geographical perspective, São Luís is characterized by extensive coastal plains, mangrove forests, *restinga* vegetation, floodplains, and beaches, as well as urban and peri-urban areas experiencing intense occupation. These coastal–estuarine environments, associated with riverine and tidal influences, create ecological mosaics that sustain a rich and heterogeneous flora, a feature that is particularly relevant for ethnobotanical studies focused on traditional knowledge and plant use [51].

In addition to its urban centrality, São Luís hosts a remarkable diversity of traditional peoples and communities, including *quilombola* communities, riverine populations, artisanal fishers, babaçu coconut breakers, and Indigenous groups occupying urban, peri-urban, and rural territories linked to estuarine and mangrove environments [51,52,53].

### 4.2. Study Design and Sampling

A descriptive-exploratory study design with an ethnopharmacological approach was adopted. A non-probabilistic convenience sampling strategy was employed. Eligibility criteria included: minimum age of 18 years, participants of both sexes, residents of the city of São Luís (MA), and users of the public healthcare system in the municipality of São Luís, Maranhão, Brazil. This approach is commonly applied in ethnographic and ethnopharmacological studies, particularly due to logistical constraints and limited access to target populations experienced during the COVID-19 pandemic [54].

Despite the non-probabilistic nature of the sample, a formal sample size calculation was performed to enhance the study’s robustness. The minimum sample size was estimated using the standard formula for prevalence studies *n* = Z^2^*p*(1 − *p*)/d^2^, as recommended by Charan and Biswas [55]. Considering previous studies reporting substantial use of medicinal plants and natural products in the population [56], an expected prevalence of 30% was adopted, with a 5% margin of error and a 90% confidence level. Under these assumptions, the minimum sample size was estimated at 228 participants. To compensate for potential losses and biases inherent in non-probabilistic sampling, the final target sample size was increased to 400 participants.

### 4.3. Instruments and Data Collection

Ethnopharmacological data were collected through a semi-structured interview (Interview S1), focusing on natural products used and/or commonly mentioned for the treatment and/or prevention of COVID-19. Participants were invited to freely list (free-listing) the medicinal plants they used against COVID-19. Additional data were collected on preparation methods, plant parts used, sources of acquisition, sources of knowledge, perceived adverse effects and contraindications, and socioeconomic information to characterize the profile of the interviewees and their health conditions during the COVID-19 pandemic (Table S1). Data collection was carried out using a hybrid approach, combining online and face-to-face interviews, between November 2022 and March 2023.

#### 4.3.1. Online Data Collection

To maximize the reach of online data collection, a network-based purposive sampling strategy, known as snowball sampling, was adopted. The online questionnaire was initially distributed via WhatsApp Messenger, version 22.24.x (Meta Platforms, Menlo Park, CA, USA) and institutional e-mail lists to undergraduate and graduate students, as well as academic and administrative staff from different programs at the Federal University of Maranhão (UFMA).

To ensure eligibility, the survey’s second question was a mandatory screening item asking whether the respondent was a user of the Brazilian Unified Health System (SUS) in São Luís. Participants who answered negatively were automatically excluded from the study and were not allowed to proceed with the questionnaire. Only individuals who confirmed prior use of public healthcare services were included in the final sample.

Recruited participants were encouraged to forward the questionnaire link within their personal networks, particularly to individuals who also met the inclusion criteria. This referral-chain dissemination strategy enabled the sample to expand beyond the researchers’ immediate academic network, reaching a broader population of users of the public healthcare system in São Luís. Online data collection was discontinued after reaching 200 valid responses.

To ensure ethical compliance, the Informed Consent Form (ICF) was presented on the first page of the questionnaire; only after acceptance were participants directed to the survey, and a complete copy of the consent form was automatically sent to the registered e-mail address.

#### 4.3.2. Face-to-Face Data Collection

Face-to-face data collection complemented the online phase and was conducted throughout the metropolitan area of São Luís (2°30′ S; 44°16′ W) between November 2022 and January 2023. Data collection sites were strategically selected as COVID-19 testing and vaccination centers, ensuring a continuous flow of the target population.

Specifically, interviews were conducted at the Primary Healthcare Units (UBS) of Cohab and São Raimundo, as well as at temporary vaccination points in major shopping centers: Shopping Rio Anil (coordinates: 2°31′30″ S, 44°15′30″ W) and Shopping da Ilha (coordinates: 2°31′35.8″ S, 44°15′38.6″ W). The inclusion of these sites enabled diversified coverage, recruiting participants in both primary healthcare settings and high-traffic public spaces, thereby enhancing the representativeness of the non-probabilistic sample. Before each interview, verbal informed consent was obtained. After the interviews, a signed informed consent form was collected.

### 4.4. Taxonomic Attribution of Ethnospecies

Among the 38 ethnospecies cited in the present study, only the most frequently mentioned species, locally known as “cat’s claw” (unha-de-gato), was collected and subjected to formal herborization and taxonomic identification. The remaining species were treated as ethnospecies, understood as traditional knowledge units based on cultural criteria and vernacular names, which do not necessarily correspond to a single taxonomically validated botanical entity [57,58].

In ethnobotanical and ethnopharmacological research, this approach is commonly adopted when systematic collection of fertile material is not logistically feasible or when the primary objective is to document local knowledge and use systems rather than to provide full taxonomic confirmation of all cited resources [59,60]. Accordingly, the ethnobotanical table presents only vernacular names (ethnospecies), following consolidated methodological practices that recognize that not all species cited in ethnodirected surveys are supported by voucher specimens, without compromising the validity of ethnocultural records [61,62]. Additionally, only the popular names of species widely recognized internationally (garlic, onion, and others) were presented in translation.

Given that the species *Uncaria tomentosa* was primarily marketed through a single establishment in the São Luís (MA) market, the establishment’s owner was contacted to obtain information on the origin of the plant material. The merchant indicated the collection area for the species, and a local expert, recognized for his experience in the field and in plant identification, guided and accompanied the collection of plant material. The specimen was collected in the municipality of Amarante do Maranhão (MA), herborized according to standard botanical procedures, and subsequently identified as *Uncaria tomentosa* (Willd.) DC by a botanist from the herbarium. The voucher specimen was deposited at the Herbarium of the Federal University of Rio de Janeiro (RFA) under accession number RFA47409.

It is noteworthy that the vernacular name “unha-de-gato” may refer to either *Uncaria tomentosa* or *Uncaria guianensis* (Aubl.) J.F. Gmel., both native to the Amazon, with overlapping distribution in Maranhão and similar medicinal uses, particularly in anti-inflammatory and immunomodulatory treatments [14,63,64]. Studies such as Silva et al. [65] highlight the coexistence of these two species in Brazilian traditional medicine and the need for morphological and chemical distinction between them.

### 4.5. Statistical Analysis

The distribution of respondents across sociodemographic profiles was evaluated using the chi-square test in SPSS for Windows, version 29.0 (IBM Corp., Chicago, IL, USA). Statistical significance was determined at α = 0.05, based on chi-square (χ^2^) values and degrees of freedom (df).

To quantitatively assess the cultural relevance, popularity, and use patterns of the recorded ethnospecies, classical ethnobotanical indices widely applied in ethnodirected research were calculated [66]. For the ethnobotanical indices (RFC, IVs, and UCs), only participants who reported using medicinal plants for the prevention or treatment of COVID-19 were included (*n* = 91). Frequency of Citation (FC) was defined as the number of informants who mentioned the use of a given species. Relative Frequency of Citation (RFC) was calculated as RFC = FC/N, where N represents the total number of medicinal plant users included in the ethnobotanical analysis, providing a standardized measure of knowledge distribution within the study population [67].

The Use Report (UR) corresponded to the total number of individual use reports attributed to each species by all informants, while the Number of Uses (NU) represented the total number of distinct use categories reported for a given species, reflecting its functional versatility. Together, these parameters allowed the assessment of both the intensity and diversity of plant use within the local medical system [67].

The Importance Value of the Species (IVS) was calculated following Byg and Balslev [68] using the formula IVS = nis/n, where nis corresponds to the number of informants who identified the species as the most important and *n* represents the total number of medicinal plant users included in the ethnobotanical analysis (*n* = 91), expressing the species’ relative prominence in the local therapeutic system. Additionally, the Use Consensus Value (UCS) was employed to assess the degree of agreement among informants regarding the usefulness of each species. UCS values range from −1 to +1 and were calculated as UCS = (2ns/n) − 1, where ns represents the number of informants who reported the use of a given species and *n* represents the total number of medicinal plant users included in the ethnobotanical analysis (*n* = 91) [68]. Higher UCS values indicate stronger consensus regarding the medicinal relevance of a given species.

### 4.6. Chemical and Biological Analyses

#### 4.6.1. Acquisition and Preparation of the Plant Material

The most referenced plant species was *Uncaria tomentosa* (Willd. ex Schult.) DC., which was collected in 2024 in the municipality of Amarante do Maranhão, MA, Brazil (geographic coordinates: −5.358537, −46.623727). The code in parentheses refers to the voucher specimen number deposited in the herbarium of the Federal University of Rio de Janeiro (RFA47409). Based on the ethnopharmacological survey, which indicated the predominant use of aqueous preparations of *U. tomentosa*, particularly decoctions, both aqueous and ethanolic extracts prepared from leaves and stem bark were included in the study. Stem bark and leaves were separately dried in a ventilated oven and ground in a hammer mill before extraction. Aqueous extracts were prepared by decoction. After extraction, the aqueous preparations were filtered and lyophilized, yielding the aqueous extracts of stem bark (AC) and leaves (AF). For the preparation of ethanolic extracts, the plant material was macerated in ethanol (1:1) for 7 days without solvent replacement. After filtration, the solvent was removed under reduced pressure using a rotary evaporator, yielding the ethanolic extracts of stem bark (EC) and leaves (EF).

#### 4.6.2. Acquisition of Fingerprints by LC-MS/MS and Dereplication Using Molecular Networking

Extracts from *Uncaria tomentosa* (EC and EF) were solubilized in acetonitrile to a final concentration of 2 mg/mL. To exclude possible suspended particles and non-solubilized material, all samples were subjected to ultracentrifugation at 10,000 RPM for 10 min. The supernatant was collected and transferred to injection vials. Next, 2 µL of each sample was analyzed using an ultra-high-performance liquid chromatography (UHPLC) system Dionex Ultimate 3000 (Thermo Fisher Scientific, Waltham, MA, USA) coupled with a unit-resolution mass spectrometer (MS) LCQfleet (Thermo Fisher Scientific, Waltham, MA, USA) with a 3D Ion Trap analyzer. The mobile phase consisted of A: water with 0.1% formic acid and 0.1% ammonium formate, and B: acetonitrile. Analyses were performed using an Acquity BEH C18 column (1.7 µL, 2.1 × 100 mm, 100 Å) with a flow rate of 0.45 mL/min, following a gradient elution as follows: 5% B from 0 to 5 min, 5–100% B from 5 to 25 min, 100% B from 25 to 30 min, 100–5% B from 30 to 31 min, and 5% B from 31 to 36 min. Samples were analyzed using an electrospray ionization (ESI) source, operating in both positive and negative ionization modes. MS spectra were acquired within an acquisition range of *m*/*z* 100–1000, with four scan events where MS^1^ signals were obtained, followed by three fragmentation events of the three most intense ions above 200 cps. The collision energy in the collision-induced dissociation (CID) cell was normalized at 35 eV. High-purity nitrogen (N_2_) was used as both dispersion and auxiliary gas. High-purity helium (He) was used as the collision gas.

The data from LC-MS analyses were converted to mzML format using ProteoWizard-MSconvert version 3.02 (ProteoWizard Development Team, Seattle, WA, USA) and processed with MZmine v.2.53 MZmine (MZmine Development Team, Brno, Czech Republic). The results from MZminealong with the converted files (.mzML) and a metadata table (containing information from biological screening tests), were uploaded to the open-source Global Natural Products Social Molecular Networking (GNPS) platform (GNPS; University of California San Diego, La Jolla, CA, USA). These files were utilized to construct molecular networks (via the FBMN workflow) and to perform compound dereplication. In addition to GNPS annotation, a custom database was employed. The parameters used followed GNPS recommendations for unit resolution data. The GNPS-generated data were then downloaded and analyzed using Cytoscape 3.9.1 (Cytoscape Consortium, San Diego, CA, USA) to obtain molecular networking results.

#### 4.6.3. Inhibition of *Spike* (RBD):ACE2 Interaction by *Uncaria tomentosa* Extracts

The ability of *Uncaria tomentosa* extracts to inhibit the interaction between the *Spike* protein’s Receptor-Binding Domain (RBD) and the Angiotensin-converting Enzyme 2 (ACE2) was evaluated by the Lumit™ SARS-CoV-2 *Spike* RBD:hACE2 Immunoassay kit (Promega, Madison, WI, USA) according to the manufacturer’s instructions and previous works from our group. Kit solutions were prepared following the provided protocol, which involves adding the extracts (250 μg/mL), then RBD and ACE2. Detection was performed using secondary antibodies conjugated to luminescent particles, which assemble to form a functional enzyme and its corresponding reactive substrate. The binding of secondary antibodies to the target proteins leads to the reconstitution of NanoBiT^®^ particles, a bioluminescence-based technology developed by Promega. Therefore, the presence of an inhibitor that blocks the interaction between RBD and ACE2 reduces the bioluminescent signal. Luminescence values were expressed as Relative Light Units (RLU) and background luminescence was corrected by subtracting the signal from blank wells (without protein–protein interaction components). Percent inhibition was then calculated from the corrected RLU values of treated samples relative to the vehicle control using the following equation:

% inhibition = [1 − (RLUtreated/RLUcontrol)] × 100

The data is the result of two assays performed in triplicate. A control experiment was performed to assess whether the extract could generate luminescence artifacts or interfere with the assay reagents (Figure S14). No residual luciferase signal was detected in the presence of the extract when compared with the blank and protein–protein interaction control conditions.

### 4.7. Ethical Parameters

This work is part of a broader project entitled “Primary Health Care, Phytotherapy and COVID-19: Reality and Expectations” approved by Plataforma Brasil through the Human Research Ethics Committee of the University Hospital Presidente Dutra Unit—HUUPD with opinion no. 5.715.855, meeting the requirements contained in the resolution no. 466/2012 of the National Health Council. The Free and Informed Consent form was signed by all research participants in two copies, initialed on every page, with one retained by the researcher and the other by the participant. Additionally, authorization for research involving biodiversity was granted by the Sistema de Autorização e Informação em Biodiversidade—SISBIO, under the license number 93412-1.

## 5. Conclusions

The present study highlights the widespread use of medicinal plants for the prevention and treatment of COVID-19 and emphasizes the role of ethnopharmacology in guiding the selection of promising species for scientific investigation. *Uncaria tomentosa* stood out for both its frequency of citation and its chemical profile, exhibiting putatively annotated oxindole alkaloids, flavonoids, iridoids, and other metabolites with potential antiviral and immunomodulatory properties. In vitro assays demonstrated that the stem bark extract was highly effective in inhibiting the interaction between the SARS-CoV-2 RBD and the ACE2 receptor, providing preliminary evidence of activity against a molecular target involved in viral entry.

The findings also reinforce the importance of adopting a cautious and scientifically rigorous approach to the therapeutic use of medicinal plants. Scientific validation, encompassing phytochemical characterization, biological assays, and clinical studies, is essential to ensure the efficacy, safety, and quality of plant-based interventions.

Due to the circumstances imposed by the COVID-19 pandemic and the social distancing measures in force during data collection, the ethnopharmacological survey relied on self-reported information obtained through a hybrid data collection strategy, which may have introduced recall bias. In addition, only *U. tomentosa* was botanically validated and subjected to chemical and biological analyses, whereas the remaining medicinal ethnospecies were recorded according to their vernacular names. Furthermore, the cross-sectional design does not allow causal inferences regarding the effectiveness of the reported therapeutic practices.

Overall, the integration of ethnopharmacological, chemical, and biological data highlights the potential of *U. tomentosa* as a source of bioactive compounds and reinforces the value of traditional knowledge as a guide for their discovery. It is hoped that these findings contribute to safer, more effective use of medicinal plants in public health contexts and support the development and strengthening of pharmacovigilance policies for herbal medicines.

## Figures and Tables

**Table 1:** Medicinal plants recorded with popular name, used parts, mode of use, frequency of citations (FC), Use Consensus Value (UCS), and relative frequency of citation (RFC).

Popular Name *	Used Parts	How to Prepare	FC	FRC	IVS	UCS
Unha-de-gato (Cat’s Claw)	Leaf	Decoction	39	0.4286	0.2387	0.8787
Boldo	Leaf	Decoction	38	0.4175	0.2307	0.8444
Limão (Lemon)	Fruit	Decoction	23	0.2527	0.0879	0.5111
Gengibre (Ginger)	Root	Maceration	22	0.2417	0.0439	0.4888
Alho (Garlic)	Bulb	Infusion	16	0.1758	0.0549	0.3555
Hortelã (Mint)	Leaf	Decoction	16	0.1758	0.0329	0.3555
Açafrão (Turmeric)	Root and rhizome	Decoction/juice	8	0.0879	0.0219	0.1777
Eucalipto (Eucalyptus)	Leaf	Decoction	7	0.0769	0.0439	0.1555
Erva-cidreira (Lemon Balm)	Leaf	Decoction	7	0.0769	0.0109	0.1555
Laranja (Orange)	Bark and flower	Decoction	6	0.0659	0.0000	0.1333
Manga (Mango)	Leaf	Decoction/Juice	4	0.0439	0.0219	0.0888
Erva-doce (Fennel)	Leaf	Decoction	4	0.0439	0.0109	0.0888
Camomila (Chamomile)	Leaf	Decoction	4	0.0439	0.0109	0.0888
Alecrim (Rosemary)	Leaf and twigs	Decoction	4	0.0439	0.0000	0.0888
Cravo-da-índia (Clove)	Flower bud	Decoction	4	0.0439	0.0000	0.0888
Mastruz (Wormseed)	Leaf/fruit	Decoction	3	0.0329	0.0109	0.0666
Cebola (Onion)	Stem	Decoction	3	0.0329	0.0000	0.0666
Urucum (Annatto)	Seeds	Decoction	3	0.0329	0.0219	0.0666
Couve (Kale)	Leaf	Decoction/juice	3	0.0329	0.0219	0.0666
Orégano (Oregano)	Leaf	Decoction	3	0.0329	0.0109	0.0666
Manjericão (Basil)	Leaf	Decoction	3	0.0329	0.0000	0.0666
Aranto	Leaf	Decoction	2	0.0219	0.0000	0.0444
Cavalinha (Horsetail)	Sterile rods	Decoction	2	0.0219	0.0000	0.0444
Canela (Cinnamom)	Bark	Decoction	2	0.0219	0.0109	0.0444
Louro	Leaf	Decoction	2	0.0219	0.0000	0.0444
Inhame	Root	Milk	1	0.0109	0.0109	0.0222
Equinacea (Coneflower)	Root and rhizome	Decoction	1	0.0109	0.0000	0.0222
Marcela	Inflorescence	Decoction	1	0.0109	0.0109	0.0222
Melão de São Caetano (Bitter Melon)	Leaf	Decoction	1	0.0109	0.0109	0.0222
Mulungu (Erythrina)	Bark	Decoction	1	0.0109	0.0000	0.0222
Capim limão (Lemon grass)	Leaf	Decoction	1	0.0109	0.0109	0.0222
Romã (Pomegranate)	Fruit	Decoction	1	0.0109	0.0000	0.0222
Malva-do-reino	Leaf	Decoction	1	0.0109	0.0000	0.0222
Goiaba (Guava)	Fruit	Juice	1	0.0109	0.0000	0.0222
Sabugueiro	Flower	Decoction/Juice	1	0.0109	0.0000	0.0222
Anis-estrelado (Star Anise)	Fruit	Decoction	1	0.0109	0.0000	0.0222

* Plants referred to by the interviewees using the regional/local vernacular name; FC = Frequency of Citation; RFC = Relative Frequency of Citation; IVS = Importance Value of Species; UCS = Use Consensus Value. Ethnobotanical indices (RFC, IVS, and UCS) were calculated considering only participants who reported the use of medicinal plants for the prevention or treatment of COVID-19 (*n* = 91). Source: the authors.

**Table 2:** LC-MS/MS data of annotated compounds in the molecular network of the ethanolic extract from the stem bark and leaves of *Uncaria tomentosa*, acquired in positive and negative ion modes.

ID	Rt (min)	Molecular Formula	[M + H]^+^ *m*/*z*	[M − H]^−^ *m*/*z*	MS/MS (MS^2^)	Proposed Compound	Stem Bark/Leaf	Ref.
**1** (**a**–**g**)	22.1	C_21_H_24_N_2_O_4_	369.2 ([M + H-H_2_O)]^+^	-	351 (-H_2_O), 337 (-CH_3_OH), 325(-CO_2_), 309 (-CH_3_OH-CO), 253, 200	oxindole alkaloids: uncarine B/C/D/E/F, mitraphylline and isomitraphylline	stem bark/leaf	[15]
**2**	28.1	C_30_H_44_O_4_	469.0	-	451 (-H_2_O), 396 (-H_2_O-C_4_H_7_)	epi-anydrobelachinal	stem bark/leaf	[16]
**3** (**a**–**b**)	13.3	C_21_H_20_O_11_	-	447.1	429 (-H_2_O), 285 (-Hex)	kaempferol-*O*-glucoside	leaf	[17]
**4** (**a**–**b**)	12.5	C_34_H_42_O_20_	-	431.2	285 (-dHex)	kaempferol-*O*-rhamnoside	stem bark/leaf	[17]
**5**	11.4	C_27_H_30_O_15_	-	593.2	431(-Hex), 285 (dHex)	kaempferol-3-*O*-rhamnosyl-galactoside	leaf	[17]
**6**	12.6	C_27_H_30_O_16_	-	609.1	447 (-Hex), 301 (-dHex)	quercetin-*O*-rhamnoside-*O*-glucoside (rutin)	leaf	[17]
**7**	12.7/12.4	C_22_H_22_O_12_	479.0	477.2	461(-H_2_O), 317 (-Hex), 175, 164	isorhamnetin-*O*-glucoside	stem bark/leaf	[18]
**8**	24.9	C_18_H_14_O_6_	-	325.3	281(-CO_2_), 263 (-CO_2_-H_2_O), 227 (-CO_2_-3H_2_O)	Uncariechin	stem bark/leaf	[19]
**9**	12.7	C_22_H_23_O_11_^+^	463.0	-	301 (-Hex), 286	peonidin 3-*O*-galactoside	leaf	[20]
**10**	19.6	C_21_H_18_O_9_	415.1	-	397 (-H_2_O), 379 (-2xH_2_O) 348 (-2xH_2_O-CH_2_OH)296	pinnatifinoside A	stem bark	[20]
**11**	14.9	C_27_H_44_O_7_	-	479.2	399, 317, 247, 209	20-hydroxyecdysone	stem bark	[21]
**12**	11.2	C_17_H_24_O_10_	-	387.2	369 (-H_2_O) 207 (-Hex) 164 (-Hex-CO) 149 (-Hex-CO-CH_3_)	Secologanin	leaf	[22]
**13**	3.9	C_16_H_24_O_10_	-	375.2	213 (-Hex), 195 (-Hex-H_2_O), 151 (-Hex-H_2_O-CO_2_)	loganic acid	leaf	[23]

## Data Availability

The datasets generated and/or analyzed during the present study are available in the Supplementary Information Files repository.

## Supplementary Materials

The following supporting information can be downloaded at https://www.mdpi.com/article/doi/s1.Figure S1: MS/MS spectrum (ESI, negative mode) of the leaf extract of *Uncaria tomentosa* with *m*/*z* 609.12; Figure S2: MS/MS spectrum (ESI, negative mode) of the leaf extract of *Uncaria tomentosa* with *m*/*z* 447.15; Figure S3: MS/MS spectrum (ESI, negative mode) of the leaf extract of *Uncaria tomentosa* with *m*/*z* 431.18; Figure S4: MS/MS spectrum (ESI, negative mode) of the leaf extract of *Uncaria tomentosa* with *m*/*z* 593.24; Figure S5: MS/MS spectrum (ESI, negative mode) of the leaf extract of *Uncaria tomentosa* with *m*/*z* 477.22; Figure S6: MS/MS spectrum (ESI, negative mode) of the leaf extract of *Uncaria tomentosa* with *m*/*z* 325.29; Figure S7: MS/MS spectrum (ESI, positive mode) of the leaf extract of *Uncaria tomentosa* with *m*/*z* 463.05; Figure S8: MS/MS spectrum (ESI, positive mode) of the leaf extract of *Uncaria tomentosa* with *m*/*z* 415.11; Figure S9: MS/MS spectrum (ESI, negative mode) of the stem bark extract of *Uncaria tomentosa* with *m*/*z* 479.22; Figure S10: MS/MS spectrum (ESI, negative mode) of the leaf extract of *Uncaria tomentosa* with *m*/*z* 387.32; Figure S11: MS/MS spectrum (ESI, negative mode) of the leaf extract of *Uncaria tomentosa* with *m*/*z* 375.22; Figure S12: MS/MS spectrum (ESI, positive mode) of the leaf extract of *Uncaria tomentosa* with *m*/*z* 369.21; Figure S13: MS/MS spectrum (ESI, negative mode) of the leaf extract of *Uncaria tomentosa* with *m*/*z* 469.04; Figure S14: Control assay evaluating potential interference of the extract with the luminescence detection system. Table S1: χ^2^ test of independence correlating descriptive variables with the use of medicinal plants for the treatment and/or prevention of COVID-19 in São Luís, Maranhão, Brazil; ICF S1. Informed Consent Form (ICF) Used in the Study; Interview S1. Semi-structured interview guide used for data collection.

## Abbreviations

The following abbreviations are used in this manuscript:

SARS-CoV-2	Severe Acute Respiratory Syndrome Coronavirus 2
WHO	World Health Organization
COVID-19	Coronavirus Disease 2019
SUS	Unified Health System
ICF	Informed Consent Form
FRC	Relative Frequency of Citation
IVS	Importance Value
FC	Number of informants who cited the species
N	Total number of informants
UCS	Use Consensus Value
NIS	Number of informants of the specific species
LC-MS/MS	Liquid Chromatography coupled to Tandem Mass Spectrometry
UHPLC	Ultra-High-Performance Liquid Chromatography
MS	Mass Spectrometry
ESI	Electrospray Ionization
CID	Collision-Induced Dissociation
GNPS	Global Natural Products Social Molecular Networking
FBMN	Feature-Based Molecular Networking
NMR	Nuclear Magnetic Resonance
RBD	Receptor Binding Domain
ACE2	Angiotensin-Converting Enzyme 2
SPR	Surface Plasmon Resonance
RdRP	RNA-dependent RNA Polymerase
CAPES	Coordination for the Improvement of Higher Education Personnel
FAPEMA	Foundation for Research and Scientific and Technological Development of Maranhão
HUUPD	University Hospital Presidente Dutra Unit
SISBIO	Authorization and Information System on Biodiversity
mzML	Mass spectrometry data format
RDA	Retro-Diels–Alder
MM-GBSA	Molecular Mechanics/Generalized Born Surface Area
