# Supplementary material for: From Traditional Knowledge to SARS-CoV-2 Entry Inhibitor Metabolites: Ethnopharmacological Investigation of Uncaria tomentosa (Willd. ex Schult.) DC
Source: Plants (Basel). 2026 Jun 27;15(13):1998. doi: 10.3390/plants15131998 (PMC13364153; doi:10.3390/plants15131998)

Supplementary material

From traditional knowledge to SARS-CoV-2 entry inhibitor metabolites: Ethnopharmacological investigation of *Uncaria tomentosa* (Willd. ex Schult.) DC

Beatriz Ribeiro Ferreira[1,2]*, Mariana Freire Campos[2]; Sarah Beatriz F. Rodrigues[3]; Ana Beatriz Lima [2]; Simony Carvalho Mendonça[2]; Crisálida M. Vilanova[3], Denise F. Coutinho[3], Diego Allonso[2]; Flavia Maria M. Amaral[3]; Suzana Guimarães Leitão[1,2].

[1] Programa de Pós-Graduação em Biotecnologia Vegetal e Bioprocessos, Universidade Federal do Rio de Janeiro, Rio de Janeiro/RJ 21941-902, Brazil.

[2] Faculdade de Farmácia, Universidade Federal do Rio de Janeiro, Rio de Janeiro/RJ 21941-902, Brazil.

[3] Departamento de Farmácia, Universidade Federal do Maranhão, Campus Bacanga, São Luís/MA 65080-805, Brazil

Table S1. χ2 test of independence, correlating descriptive variables with the use of plant species employed and/or popularly referred to in the treatment and/or prevention of COVID-19 in São Luís, Maranhão, Brazil.

Variables	Use of Plants in COVID-19				Total	
	Yes		No			
	N	%	N	%	N	%
**Sex**						
Male	21	17,36	100	82,64	121	30,25
Female	70	25,09	209	74,91	279	69,75
**Total**	91	22,75	309	77,25	400	100.00
**Age (years)** [a]						
≤ 18	0	0	13	100	13	3,25
18-29	31	14,90	177	85,10	208	52,00
30-59	42	30,66	95	69,34	137	34,25
≥ 60	18	42,86	24	57,14	42	10,50
**Total**	91	22,75	309	77,25	400	100,00
**Education**						
Illiterate	0	0	1	100	1	0,25
Incomplete primary education	7	38,89	11	61,11	18	4,50
Complete primary education	2	18,18	9	81,82	11	2,75
High school incomplete	1	14,29	6	85,71	7	1,75
Completed high school	39	23,49	127	76,51	166	41,50
Higher education incompleted	2	100	0	0	2	0,50
Higher education completed	21	16,94	103	83,06	124	31,00
Postgraduate studies	19	26,76	52	73,24	71	17,75
**Total**	91	22,75	309	77,25	400	100,00
**Income**						
< 2 minimum wages	39	27,86	101	72,14	140	35,00
2-4 minimum wages	30	18,52	132	81,48	162	40,50
5-10 minimum wages	17	22,08	60	77,92	77	19,25
> 10 minimum wages	5	23,81	16	76,19	21	5,25
**Total**	91	22,75	309	77,25	400	100,00

a: $p<0{,}001$ Source: the author.

ICF S1. Informed Consent Form (ICF) Used in the Study

UNIVERSIDADE FEDERAL DO MARANHÃO - UFMA

PROGRAMA DE PÓS GRADUAÇÃO EM SAÚDE E AMBIENTE

FITOTERAPIA COMO TERAPIA ALTERNATIVA E COMPLEMENTAR NA COVID-19: REALIDADE E EXPECTATIVA

O senhor(a) está sendo convidado a participar da pesquisa intitulada “Fitoterapia como terapia alternativa e complementar NA COVID-19: realidade e expectativa”, a ser realizada pela Mestranda Beatriz Ribeiro Ferreira, sob a orientação da Profª Drª Flavia Maria Mendonça do Amaral, da Universidade Federal do Maranhão, que tem como objetivo principal realizar um estudo de caracterização das espécies vegetais empregadas e/ou referidas popularmente na terapêutica e/ou prevenção da COVID-19; bem como investigar as informações divulgadas em sites nacionais para tais fins.

Nessa etapa da pesquisa, será realizado um levantamento de dados através da aplicação de uma entrevista semiestruturada com foco na abordagem das espécies vegetais empregadas e/ou referidas popularmente na terapêutica e/ou prevenção da COVID-19. Além disso será realizada uma revisão sistemática no site de busca Google, divulgados a partir de dezembro de 2019. Para no fim obtermos um banco de dados completo e usual.

Assegura-se que a identidade dos participantes será confidencial e que os dados coletados serão utilizados exclusivamente para atender aos objetivos da pesquisa. A conduta de coleta será através de uma entrevista, o qual foi elaborado sem inclusão de perguntas pessoais e/ou constrangedoras; portanto espera-se que os (as) entrevistados (as) não sofram riscos ou danos morais e/ou pessoais. Mas, embora com o cuidado na elaboração desse instrumento, pode sim, ocorrer de algum entrevistado não se sentir à vontade ou constrangido para responder algum item contemplado na entrevista. Sendo assim, o(a) senhor(a) tem a liberdade total de recusar a participação ou retirar seu consentimento em qualquer fase da pesquisa. Ressalta-se também que o (a) entrevistado(a) não terá qualquer custo financeiro diante da pesquisa.

Entre os benefícios da pesquisa, o desenvolvimento do estudo etnofarmacológico aqui proposto, possibilitará obtermos dados da(s) forma(s) de preparação(ões), a(s) parte(s) utilizada(s), forma de obtenção, forma de preparação,

origem da informação, cuidados na guarda e conservação da reparação, conhecimento sobre possíveis efeitos colaterais e contraindicação, além dos dados sócio-econômicos; possibilitando, assim, a caracterização do uso de espécies vegetais empregadas terapeuticamente em idosos na amostra em estudo.

A análise desses dados permitirá ampliar o conhecimento acerca das espécies vegetais que podem ser utilizadas como alternativa e/ou complemento, quer na terapêutica e/ou na prevenção de COVID-19, para que assim consigamos alinhar se existe a prospecção de um possível bioproduto de origem vegetal para o enfrentamento desta pandemia, a partir das espécies em uso.

Os resultados poderão ser publicados em revistas da área da saúde ou interdisciplinar assim como apresentados em simpósios e/ou congressos.

Garante-se ainda que, em qualquer etapa do estudo, os participantes terão acesso aos responsáveis pela pesquisa para esclarecimento de eventuais dúvidas. Ficam disponíveis os contatos da pesquisadora responsável Beatriz Ribeiro Ferreira e Professora Dra. Flavia Maria Mendonça do Amaral, da Universidade Federal do Maranhão. Havendo dúvidas, questionamentos e/ou denúncias, registra-se o endereço e telefone do Comitê de Ética e Pesquisa da Universidade Federal do Maranhão, situado na Sala 07, Bloco C, CEB Velho, Campus Universitário do Bacanga da UFMA (fone: 2109-8708).

gov.br Documento assinado digitalmente
BEATRIZ RIBEIRO FERREIRA
Data: 17/11/2022 09:24:30-0300
Verifique em https://verificador.iti.br

gov.br Documento assinado digitalmente
FLAVIA MARIA MENDONCA DO AMARAL
Data: 17/11/2022 13:02:45-0300
Verifique em https://verificador.iti.br

Beatriz Ribeiro Ferreira
(98)981783002

Flavia Maria Mendonça do Amaral
(98)981147738

Tendo recebido todas as informações necessárias, eu, _____________________________RG n° __________________________, aceito de livre e espontânea vontade, participar dessa pesquisa, e informo que assinei e recebi a cópia deste documento.

São Luís, _____ de __________________ de 2022.

Interview S1. Semi-structured interview guide used for data collection

INTERVIEW

Are you a user of the public health system in the municipality of São Luís, Maranhão?

( ) Yes ( ) No

(If yes, proceed to the next questions)

1. **Name**:____
2. **Sex**: ( ) F ( ) M
3. **Age:** ________ year
4. **Neighborhood of residence:**__________________________________
5. **Education level:**
   ( ) Illiterate
   ( ) Incomplete primary education
   ( ) Complete primary education
   ( ) Incomplete secondary education
   ( ) Complete secondary education
   ( ) Incomplete higher education
   ( ) Complete higher education
   ( ) Postgraduate degree
6. **Family income bracket:**
   ( ) Less than 2 minimum wages
   ( ) 2 to 4 minimum wages
   ( ) 5 to 10 minimum wages
   ( ) More than 10 minimum wages
7. **Do you have any health conditions?** ( ) Yes ( ) No
8. **If yes, which ones**
   ( ) Arterial Hypertension ( ) Diabetes
   ( ) Hypercholesterolemia ( ) Nephropathies
   ( ) Obesity ( ) Migraine/Headache
   ( ) Osteoporosis
   ( ) Other:____
9. **Did you have COVID-19?** ( ) Yes, confirmed by a test.
   ( ) No, confirmed by a test.
   ( ) I don't know. I had symptoms but did not get tested.
   ( ) I don't know. I had no symptoms and did not get tested.
10. **Do you use (or have you ever used) any plant for the treatment and/or prevention of COVID-19?**
    ( ) Yes ( ) No

(If the answer to question 10 is NO, proceed with questions 11A, 11B, and 11C)

11A. If you answered NO to the use of any plant for the treatment and/or prevention of COVID-19: why did you not use one?

( ) Not familiar with any

( ) Do not believe in it

( ) Difficulty accessing them

( ) Only use medications prescribed by a doctor

( ) Other: ____

11B. Even though you have not used any, are you aware of any plant recommended for the treatment and/or prevention of COVID-19?

( ) Yes ( ) No

11C. If yes, which plant(s)?

____
____

(If the answer to question 10 is YES, proceed with the questions below regarding the characterization of plant use in the treatment and/or prevention of COVID-19)

12. **Which plant(s) do you use (or have you used) for the treatment and/or prevention of COVID-19, and which part(s) of the plant(s) were used?**
____
____

13. **How did you obtain information about plants used in the treatment and/or prevention of COVID-19?**
( ) Family/friends
( ) Healthcare professional (doctor, pharmacist, nurse)
( ) Media (television, radio, internet, leaflets, newspapers, or magazines)
( ) Other:____

14. **Where did you obtain the plant(s) used in the treatment and/or prevention of COVID-19?**
( ) Home garden
( ) Pharmacy/drugstore
( ) Open market/marketplace
( ) Natural products store
( ) Other:____

15. **What preparation method did you use (or do you use) medicinally for these plants?**
( ) Decoction: Place the plant in a container, add cold water, and boil for 10 minutes with the container covered.
( ) Infusion: Pour boiling drinking water over the herb in a cup and steep covered.
( ) Maceration: Grind the plant and add a solvent (water or other).
( ) Herbal tonics (Garrafadas)
( ) Compresses
( ) Ointments
( ) Other:____

16. **What dosage was used (how many times per day did you or do you take it)?**

( ) Once ( ) Twice
( ) Three times ( ) Other:___________________________________

17. For how long: ( ) days ( ) weeks

18. What is your level of satisfaction with the treatment?

( ) Excellent
( ) Good
( ) Fair
( ) Poor
( ) No effect

19. The last time you used medicinal plants for the treatment and/or prevention of COVID-19, did you experience any adverse effects? ***(You may select more than one option)***

( ) No effect ( ) Headache ( ) Dizziness
( ) Heart palpitations ( ) Heartburn
( ) Abdominal cramps ( ) Bitter/metallic taste in the mouth
( ) Indigestion ( ) Nausea
( ) Other:____

20. Have you ever heard any information about possible risks, toxicity, or harmful effects associated with the use of medicinal plants for the treatment and/or prevention of COVID-19?

( ) Yes ( ) No

20.1 If yes, which? ____

21. During the period in which you used the medicinal plant, did you simultaneously use any synthetic medication for the treatment of other conditions (such as diabetes, hypertension, etc.)?

( ) Yes ( ) No

21.1 If yes, which? ____

22. If you answered "yes" to question 20, did you inform your doctor and/or other healthcare professionals about the use of these medicinal plants during the treatment and/or prevention of COVID-19?

( ) Yes ( ) No

Figure S1. MS/MS spectrum (ESI, negative mode) of the leaf extract of *Uncaria tomentosa*, with *m/z* 609.12 [M-H]$^-$

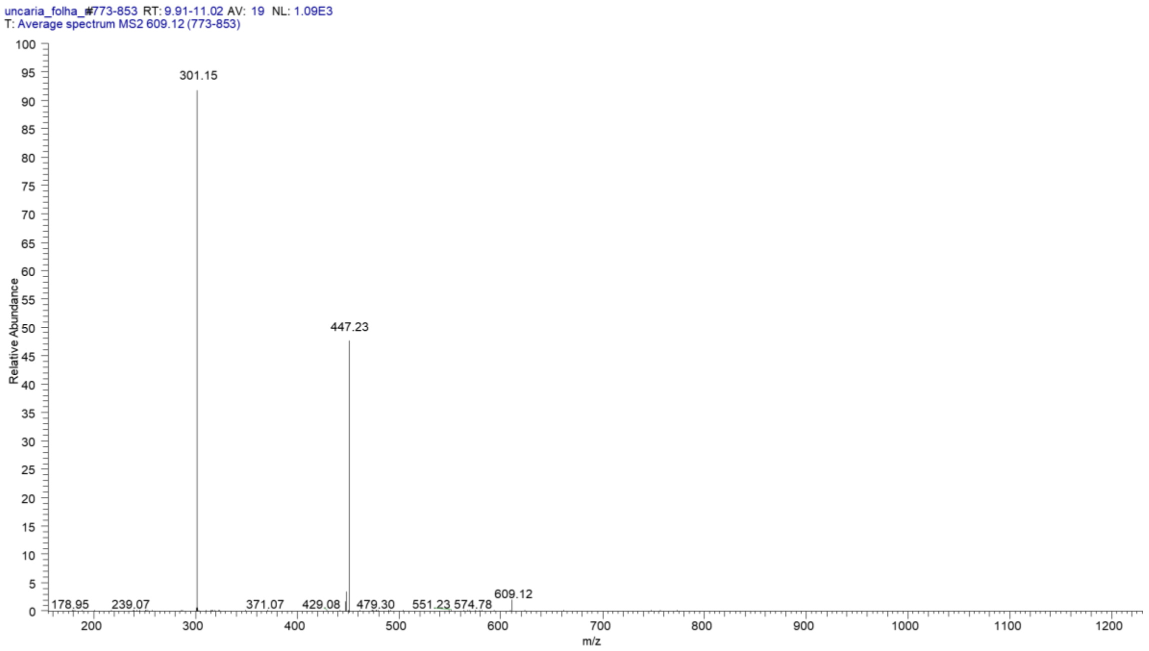

Figure S2. MS/MS spectrum (ESI, negative mode) of the leaf extract of *Uncaria tomentosa*, with *m/z* 447.15 [M-H]$^-$

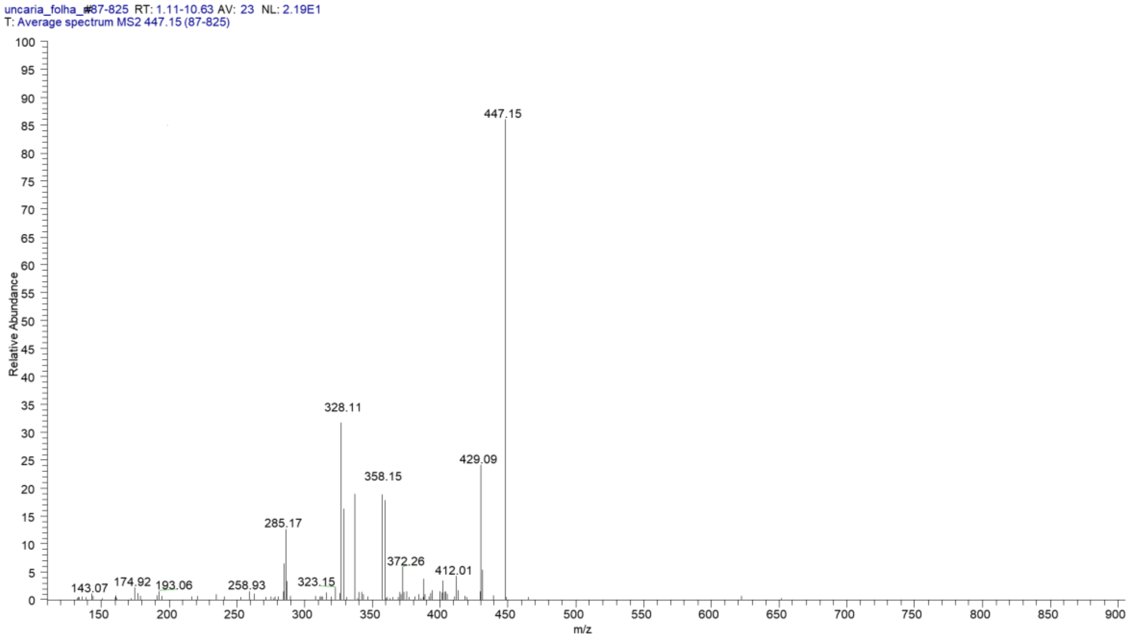

Figure S3. MS/MS spectrum (ESI, negative mode) of the leaf extract of *Uncaria tomentosa*, with *m/z* 431.18 [M-H]$^-$

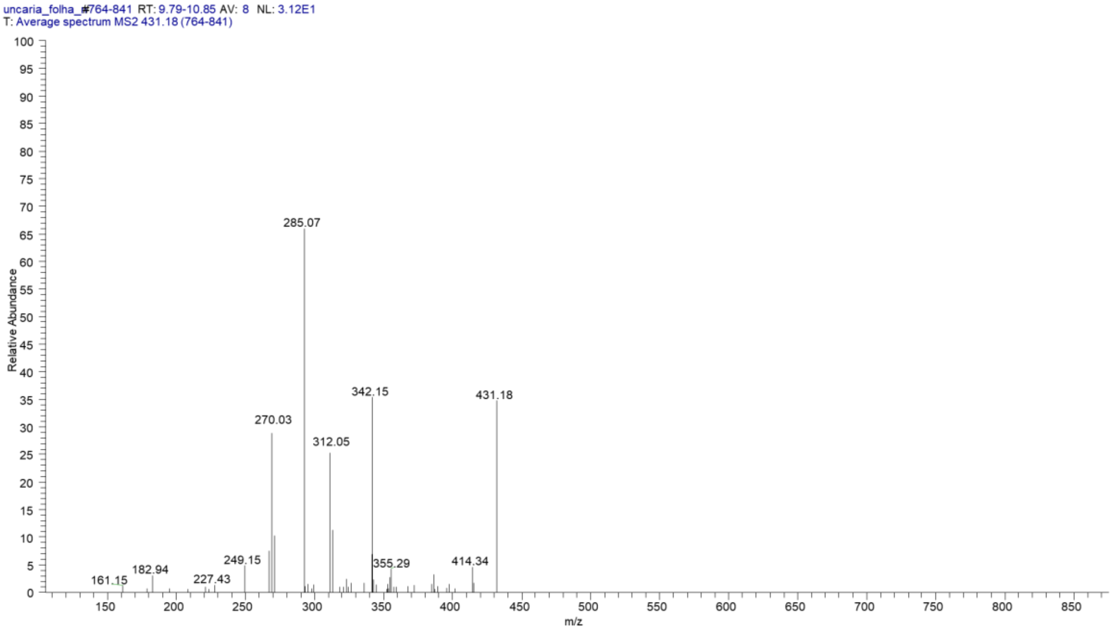

Figure S4. MS/MS spectrum (ESI, negative mode) of the leaf extract of *Uncaria tomentosa*, with *m/z* 593.24 [M-H]$^-$

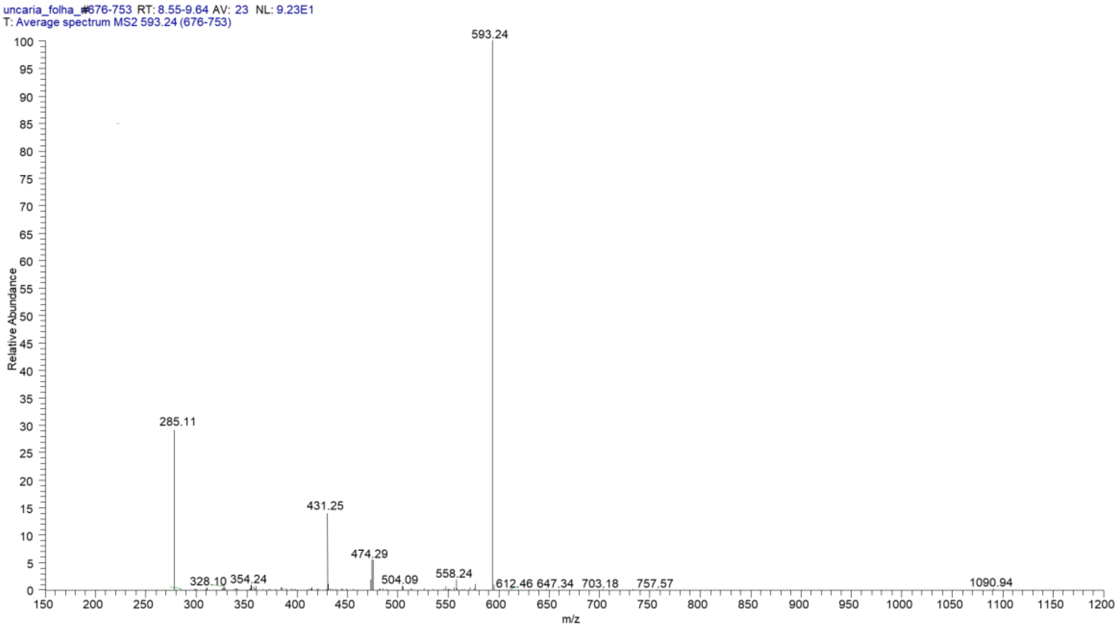

Figure S5. MS/MS spectrum (ESI, negative mode) of the leaf extract of *Uncaria tomentosa*, with *m/z* 477.22 [M-H]$^-$

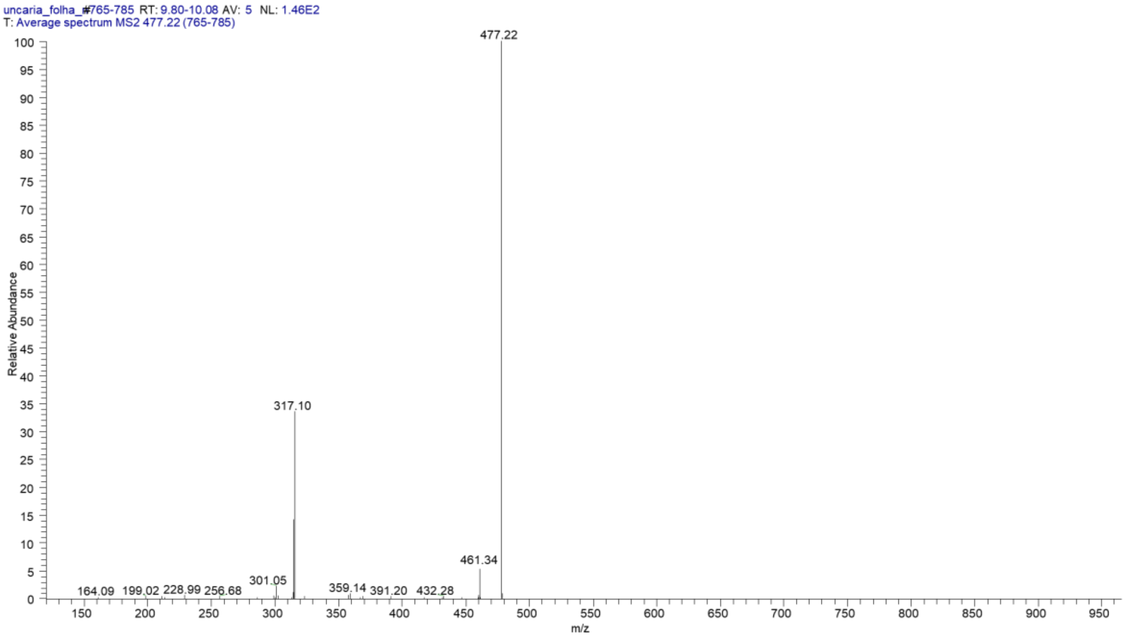

Figure S6. MS/MS spectrum (ESI, negative mode) of the leaf extract of *Uncaria tomentosa*, with *m/z* 325.29 [M-H]$^-$

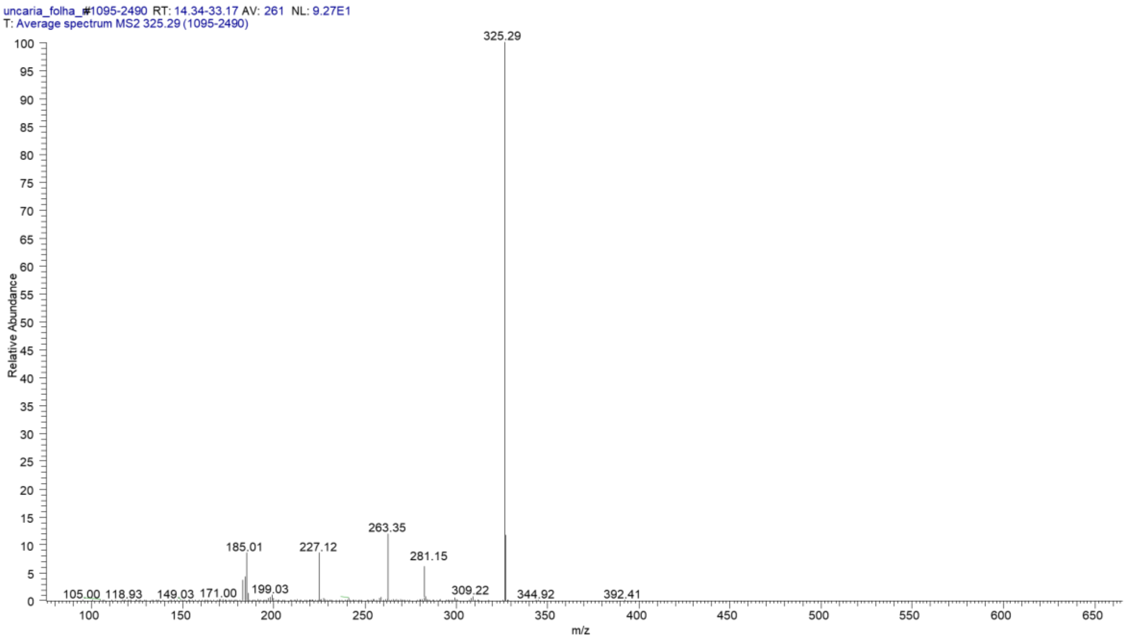

Figure S7. MS/MS spectrum (ESI, positive mode) of the leaf extract of *Uncaria tomentosa*, with *m/z* 463.05 [M+H]$^+$

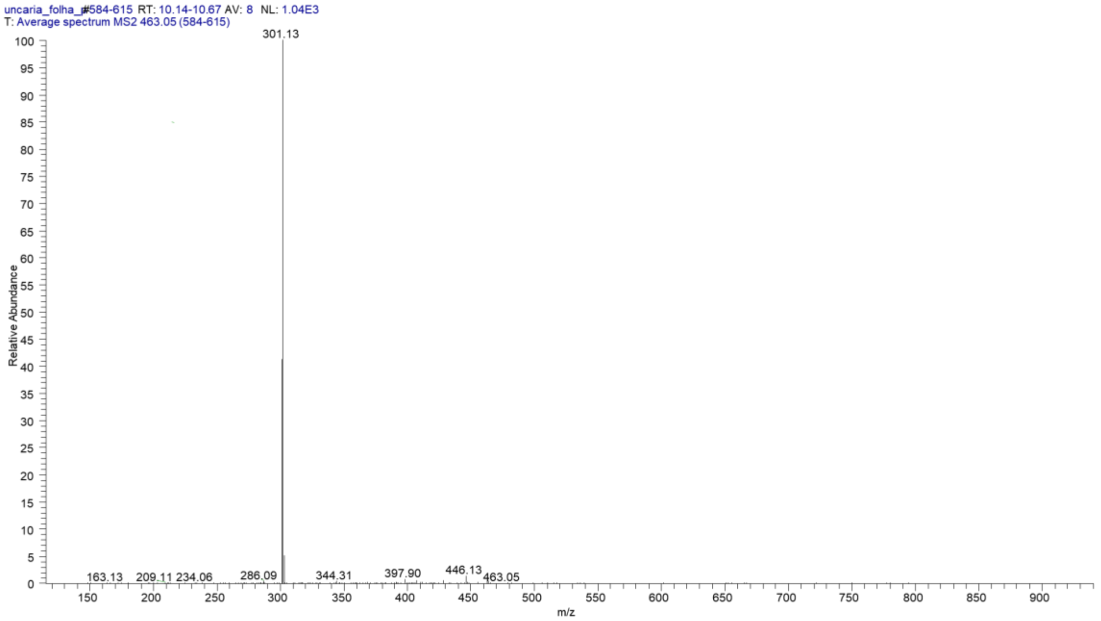

Figure S8. MS/MS spectrum (ESI, positive mode) of the leaf extract of *Uncaria tomentosa*, with *m/z* 415.11 $[M+H]^+$

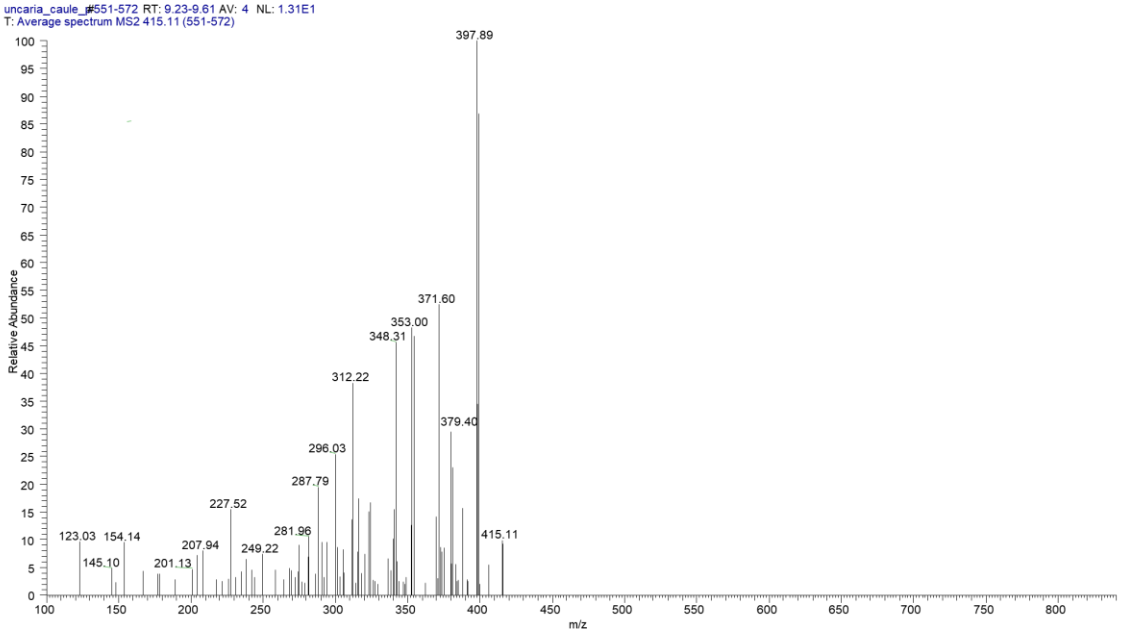

Figure S9. MS/MS negative (ESI, negative mode) of the steam bark extract of *Uncaria tomentosa*, with *m/z* 479.22 $[M-H]^-$

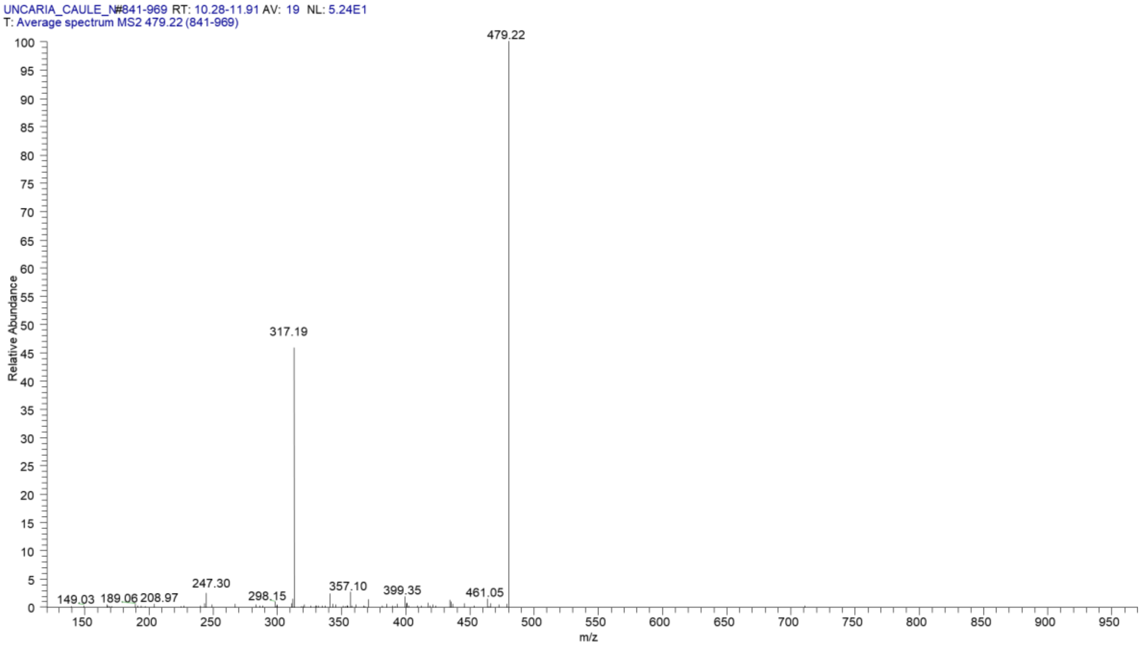

Figure S10. MS/MS negative (ESI, negative mode) of the leaf extract of *Uncaria tomentosa*, with *m/z* 387.32 [M-H]$^-$

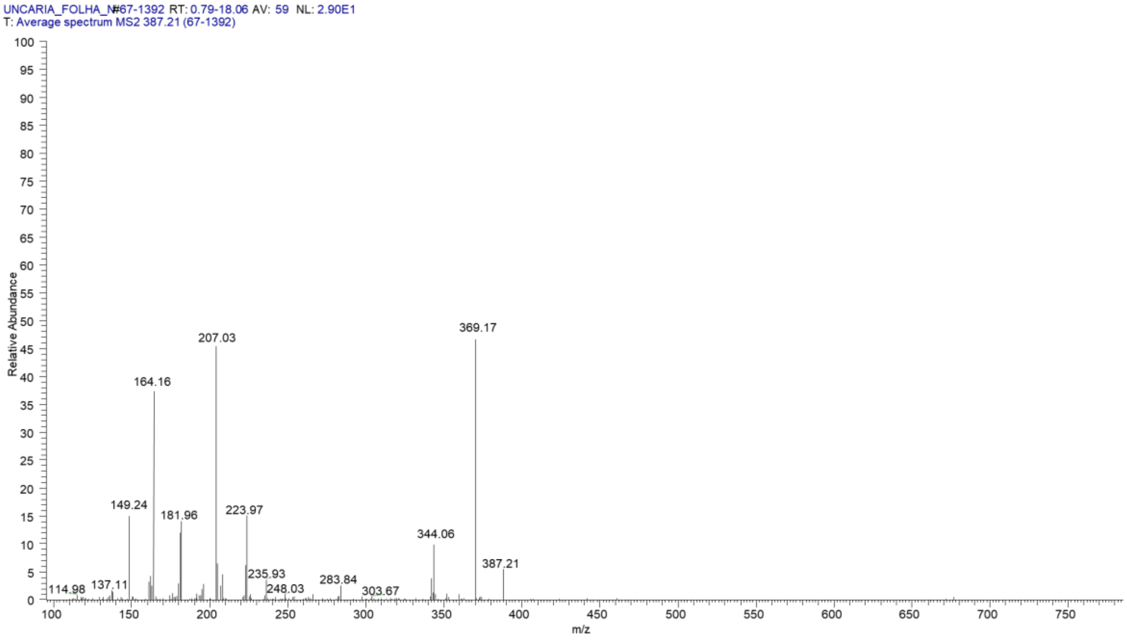

Figure S11. MS/MS negative (ESI, negative mode) of the leaf extract of *Uncaria tomentosa*, with *m/z* 375.22 [M-H]$^-$

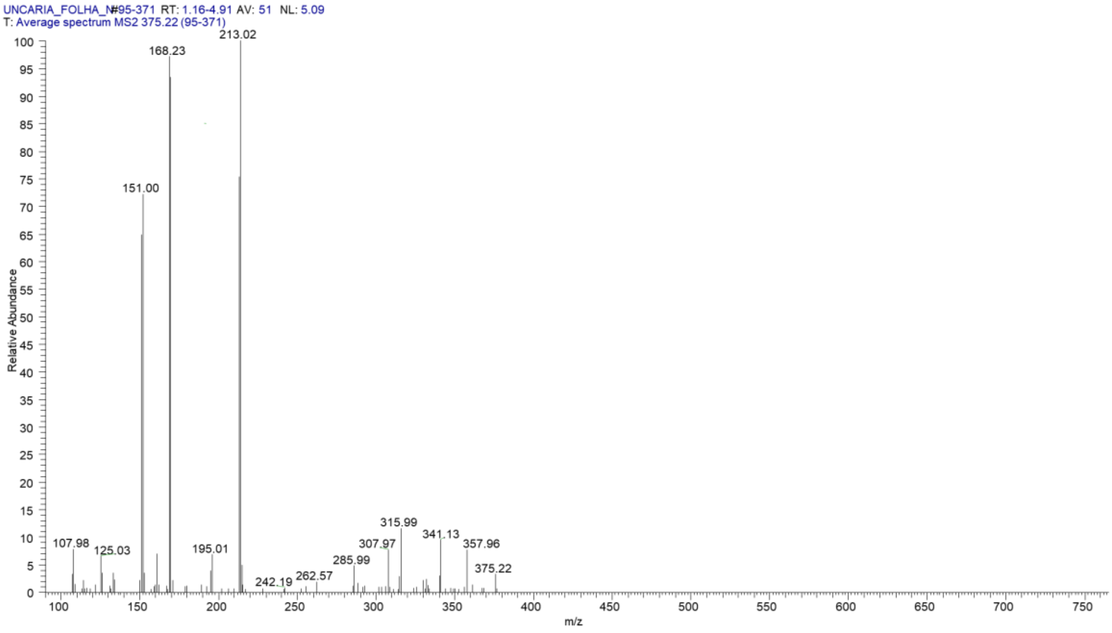

Figure S12. MS/MS negative (ESI, positive mode) of the leaf extract of *Uncaria tomentosa*, with *m/z* 369.21 $[M+H]^+$

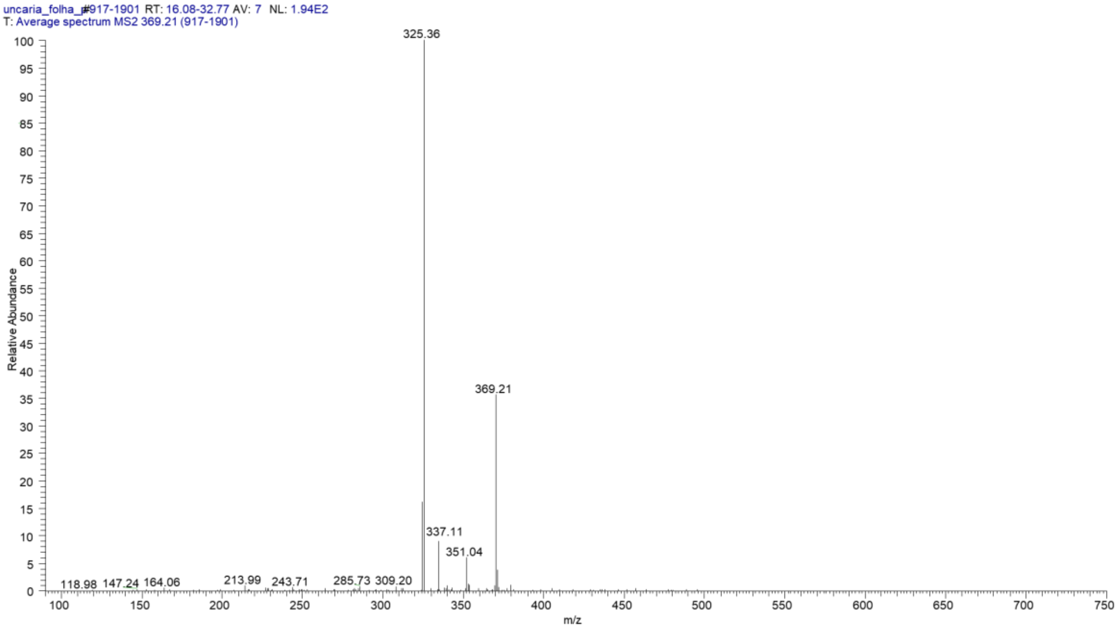

Figure S13. MS/MS spectrum (ESI, positive mode) of the leaf extract of *Uncaria tomentosa*, with *m/z* 469.04 $[M+H]^+$

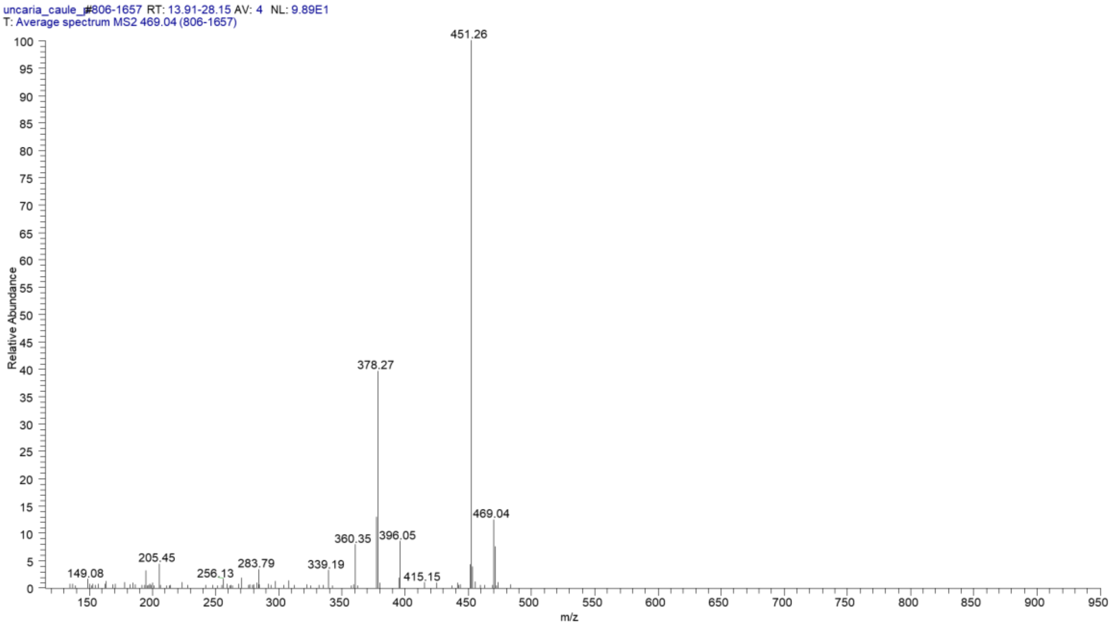

Figure S14. Control assay assessing potential interference of U. tomentosa stem bark extractt with the Lumit® components. No detectable luminescence signal was observed.

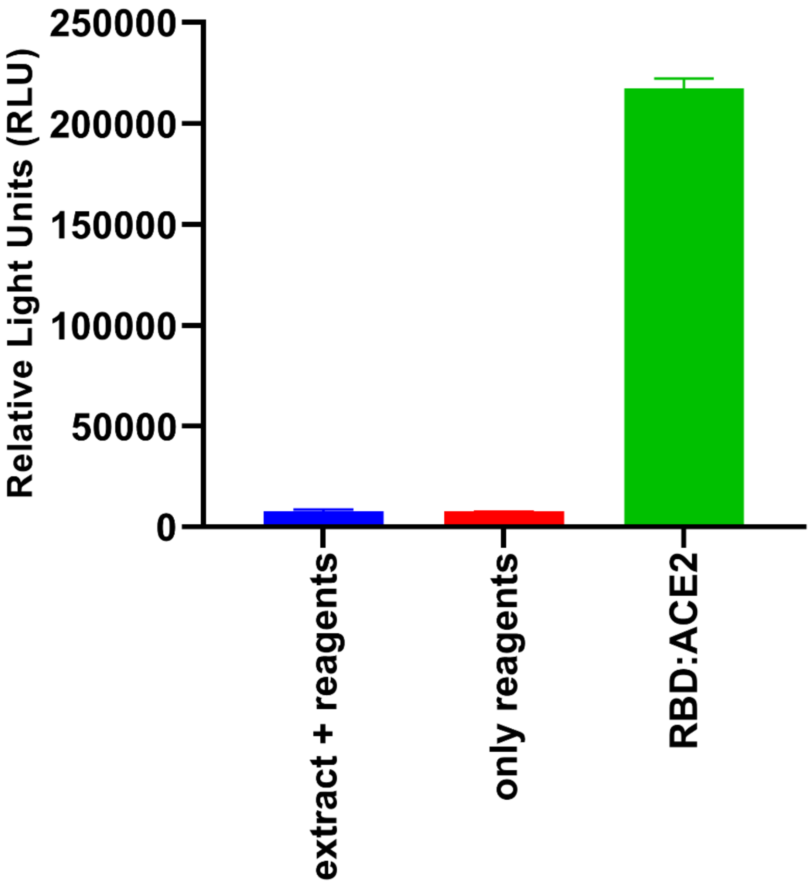

Supplement: Supplementary file 1 [file plants-15-01998-s001.zip › plants-4351029-supplementary.pdf]
